# The Effect of Ag-Decoration on rGO/Water Nanofluid Thermal Conductivity and Viscosity

**DOI:** 10.3390/nano12071095

**Published:** 2022-03-26

**Authors:** Felipe Lozano-Steinmetz, Victor A. Martínez, Diego A. Vasco, Alonso Sepúlveda-Mualin, Dinesh Patrap Singh

**Affiliations:** 1 Department of Mechanical Engineering, Faculty of Engineering, University of Santiago of Chile (USACH), Av. Lib. Bdo. O’Higgins 3363, Estación Central, Santiago 9170022, Chile; felipe.lozano@usach.cl (F.L.-S.); victor.martinezg@usach.cl (V.A.M.); 2 Millennium Institute for Research in Optics (MIRO), Physics Department, Faculty of Science, University of Santiago of Chile (USACH), Av. Víctor Jara 3493, Estación Central, Santiago 9170124, Chile; alonso_sepulveda@hotmail.com (A.S.-M.); singh.dinesh@usach.cl (D.P.S.)

**Keywords:** nanofluids, reduced graphene oxide, Ag decoration

## Abstract

Carbon-based nanomaterials have a high thermal conductivity, which can be exploited to prepare nanofluids. Graphene is a hydrophobic substance, and consequently, graphene-based nanofluid stability is improved by adding surfactants. An attractive alternative is the decoration of reduced graphene oxide (rGO) with metallic materials to improve the thermal conductivity without affecting the stability of nanofluids. This study focuses on the synthesis and characterization of rGO/Ag (0.1 wt.%) aqueous nanofluids. Moreover, the effects of the Ag concentration (0.01–1 M) on the thermal conductivity and viscosity during the synthesis of rGO/Ag composite are analyzed. The nanofluid thermal conductivity showed increases in relation to the base fluid, the most promising being 28.43 and 26.25% for 0.1 and 1 M of Ag, respectively. Furthermore, the nanofluids were Newtonian in the analyzed range of shear rates and presented a moderate increase (<11%) in viscosity. Aqueous nanofluids based on rGO/Ag nanocomposites are a potential alternative for applications as heat transfer fluids.

## 1. Introduction

The incorporation of nanomaterials into fluids used in the refrigeration industry, such as water, oils, and ethylene glycol, has become a promising alternative to improve heat transfer in several applications. These nano-modified working fluids are commonly named nanofluids after the work of Choi and Eastman [1]. Over the years, researchers have used nanoparticles with varying chemical natures to increase the heat transfer capacities of fluids, namely metals, metallic oxides, and, more recently, carbon-based nanomaterials due to their lower density and higher thermal conductivity.

The modification of fluids with carbon-based nanomaterials provides working fluids with better capabilities, with potential applications in renewable and clean energy technologies, such as solar energy, geothermal energy, and electronic cooling [2,3,4]. As an example of this, Mehrali et al. [5] found that nanofluids based on graphene decorated with silver can be used in the direct absorption of solar radiation in solar collectors during short periods, since they achieve efficiencies of 77%.

In electronic cooling applications, Selvaraj et al. [6] studied nanofluids made of graphene nanocomposites and Al${}_{2}$O${}_{3}$. The authors found a maximum thermal conductivity increase of 45%, and maximum increases in the dimensionless Nusselt number (Nu) and the coefficient of convective heat transfer of 16% and 51.7%, respectively, with a concentration of 0.2 vol.%. Recently, a novel application for nanofluids based on graphene and rGO using both aqueous and organic electrolytes was reported.

These nanofluids, called electroactive nanofluids, have demonstrated potential applications in energy storage, specifically in flow cells. The electroactive nanofluids are stored out of the electrochemical cell and are forced to circulate through it by pumping [7].

Carbon-based nanomaterials are available in different nanostructures, such as graphene (G), graphene nanoplatelets (GNP), graphene quantum dots (GQDs), multi-layer graphene oxide (MLG), and graphene oxide (GO) and its reduced form (rGO) [8]. Among them, a widely studied material is graphene oxide, which is made out of graphene sheets via Hummers’ method straight from graphite powder. Esfahani et al. [9] revealed increases in thermal conductivity of 8.7% and 18.9% at 25 °C when raising the GO concentration to 0.01 wt.% and to 0.1 wt.%, respectively.

Furthermore, the authors discovered that water-based GO nanofluids exhibited non-Newtonian behavior (shear thinning) at lower shear rates and that the viscosity increased linearly as the GO concentration rose from 0.01 to 0.1 wt.%. Ijam et al. [10] studied GO water/EG nanofluid viscosity. The nanofluid displayed shear-thinning and Newtonian behavior at low and high shear rates, respectively. Furthermore, at 0.1 wt.% and 20 °C, the nanofluid viscosity increased by 35% against the base fluid and exhibited shear-thinning behavior at low shear rates. As the shear rate increased, the aggregate particles began to disintegrate and to align in the shear flow direction, causing the viscosity to decrease and reach a Newtonian plateau at high shear rates.

To generate more stable solutions, the alternative of reduced graphene oxide (rGO) was raised. This nanomaterial is a two-dimensional layered material with a one-atom-thick, honeycomb structure, which is obtained by reducing the oxygen of the graphene oxide (GO). Said et al. [11] demonstrated that a hybrid mixture of functionalized carbon nanofibers/rGO nanofluid (0.04 vol.%) showed outstanding stability by measuring the zeta potential. Zhang et al. [12] studied controlled rGO nanofluids that showed good stability with increased temperature and additive concentration. The authors obtained significant thermal conductivity enhancements in rGO/DI-water nanofluids reaching a maximum enhancement of 32.19% at 60 °C with 1.0 mg/mL of rGO.

According to Kamitchi et al. [13], there was a 10% increase in the thermal conductivity for rGO/DI-water nanofluids (0.3 mg/mL) at 75 °C. Recently, Pavia et al. [14] presented a review article focused on the thermal conductivity of graphene-based nanofluids. Specifically, the authors showed that the thermal conductivity enhancement at 30 °C of rGO-based aqueous nanofluids ranged from 9 to 18%, depending on the rGO weight fraction. Mandhare et al. [15] prepared an aqueous nanofluid with a rGO-ZnO nanocomposite.

The authors found that the thermal conductivity of the nanofluid increased from 0.649 to 1.643 W/mK (153%) and 0.796 to 2.95 W/mK (271%) for a temperature rise from 34 to 50 °C for rGO-ZnO nanocomposite concentrations of 0.03 and 0.1 vol.%, respectively. Chawhan et al. [16] prepared and characterized a water-based nanofluid with rGO-SnO${}_{2}$ nanocomposites of different rGO-SnO${}_{2}$ mass ratios (1:7, 1:8, and 1:10). The authors found that the increase in the volumetric concentration of rGO-SnO${}_{2}$ raised the thermal conductivity of the nanofluid for all the mass ratios analyzed, and this effect was more noticeable at higher temperatures.

Further, the prepared nanofluid exhibited non-Newtonian pseudoplastic rheological behavior in the shear rate range of 0–4000 s${}^{-1}$. Nevertheless, the rheology of rGO/water-based nanofluids has shown similar behavior to those prepared with GO-namely, at high shear rates, a Newtonian behavior [12,17,18]. In their review on the rheological properties of graphene-based nanofluids, Hamze et al. [19] agreed that rGO-based aqueous nanofluids tend to be Newtonian at high shear rates (>100 s${}^{-1}$) and low volumetric concentrations (<0.01 vol.%).

GNP are multi-layer graphene nanoplatelets made up of 10–30 graphene sheets with a high degree of graphene property retention [8], a lower cost than carbon nanotubes [20], and easier dispersion than graphene or carbon nanotubes. Nevertheless, the GNP thermal conductivity decreases with the number of layers [21].

Yarmand et al. [22] found that thermal conductivity was enhanced by 13.56% (20 °C) and 15.87% (40 °C), for a functionalized-GNP/water-based nanofluid with 0.1 wt.%. In addition to other carbon-based nanomaterials, the addition of GNPs increased the viscosity of the base fluids. The same researchers also [23] studied the viscosity of functionalized GNP and Pt-decorated GNP water-nanofluids, showing 24% and 33% increases at 0.1 wt.% and 313 K, respectively.

Despite the significant progress made in nanofluid synthesis with carbon-based nanomaterials, in polar fluids, graphene does not disperse well since it is a hydrophobic substance. As a result, adding surfactants to graphene-based nanofluids improves their stability [24,25]. Although the incorporation of surfactants in nanofluids of carbon-based nanomaterials has demonstrated improving their stability [26], surfactants may decrease the performance of these nanofluids through the reduction in their thermal conductivity [27,28], the low-temperature degradation of surfactants, and their tendency to produce foam, which implies additional thermal resistance [29,30].

Several chemical functionalization methods exist that can provide long-term stability to nanofluids. Among them, researchers have used free-surfactant dispersion techniques, which generally involve treating the nanomaterial with an acid or alkaline medium [31]. This is the case of Baby et al. [32], who used an acid treatment in DI-water and ethylene glycol to exfoliate graphene and functionalize it. Despite both nanofluids showing good stability, the thermal conductivity exhibited a higher increase in aqueous nanofluids (75% with 0.05 vol.%) than in EG-based nanofluids (5% with 0.08 vol.%). The authors suggested that the higher viscosity of EG explains this remarkable difference.

Decoration of the nanostructure surface of carbon-based nanomaterials is another approach to reducing their agglomeration tendency, maintaining their capability to improve thermal conductivity [33,34]. The decoration of the nanostructure is possible since graphene-oxide sheets have functional groups attached to their surface, generating synergic effects in several applications [35,36,37]. Baby et al. [38] produced a graphene-based nanocomposite formed by coating 20% of the f-HEG surface with CuO through chemical reduction. The DI water-based nanofluid displayed a rising thermal conductivity of almost 90% at 50 °C with 0.05 vol.%.

The authors found that using EG as base fluid, the thermal conductivity enhancement was lower, reaching 23% at 50 °C for 0.07 vol.%. The authors suggest that the functional groups added to the HEG during the acid treatment may benefit decorating metal/metal oxide nanoparticles on their surface, which remain in the graphene nanosheet’s surface even after the ultrasonication process. Considering the growing use of silver nanoparticles as a supporting material for the synthesis of nanocomposite materials [39,40], the same team [41] decorated the surface of f-HEG with Ag nanoparticles with the help of carboxyl and hydroxyl functional groups present in HEG.

Ag/HEG was suspended separately in DI water as well as EG without a surfactant. The thermal conductivity of DI water nanofluids achieved improvements of 25% at 25 °C and 86% at 70 °C with 0.05 vol.%. However, the EG-based nanofluids did not exhibit any significant increase in their thermal conductivity until 0.05 vol.%. This difference was attributed to the high viscosity of the base fluid and the low particle-particle interaction. To study the decoration degree, Gu et al. [42] synthesized several acid-treated CNT-based nanocomposites through the decoration of different loadings of silver acetate ranging from 0.1 to 1.0 mol%.

Ag nanoparticles formed on the surface of the CNT, and their size increased with silver decoration concentration, registering an average size of 50 nm with 0.1 mol% and 80 nm for 1.0 mol%. The authors suggest that this phenomenon may increase the distance between CNTs, thus, helping to prevent aggregation. Regarding viscosity, the authors found that the Ag decoration fraction decreases the viscosity magnitude of distilled water-based nanofluids synthetized with 3 wt.%. Additionally, the thermal conductivity of the samples was registered at 20 °C, and nanocomposite weight fractions ranging from 0 to 3.0 wt.%. Moreover, the Ag decoration loading increased the thermal conductivity enhancement up to 21% at 3.0 wt.% and 1.0 mol% of Ag.

Compared with covalent methods, modification by non-covalent methods allows for preservation of the graphene sheets’ properties, and the stability of the prepared nanofluids is enhanced [8]. Decoration with metals and metallic oxides has emerged as an attractive alternative to the addition of surfactants since decoration may preserve the thermal conductivity improvement of graphene without affecting the stability of the nanofluids [43]. According to our knowledge, the preparation and characterization of rGO/Ag nanofluids have not been studied. Another contribution is the analysis of the effect of Ag concentration during the preparation of the decorated rGO on the thermal conductivity and viscosity of the prepared nanofluids. All the water-based rGO/Ag nanofluids were prepared with a concentration of 0.1 wt.% based on similar studies that showed significant thermal conductivity improvements by using rGO with this concentration [9,40,44].

## 2. Materials and Methods

### 2.1. Synthesis of Ag-Decorated Graphene

The microwave reduction method allowed for the synthesis of expanded graphite. First, 6 g of graphite powder was mixed in a solution mixture of H${}_{2}$SO${}_{4}$ (150 mL) and H${}_{3}$PO${}_{4}$ (50 mL) under constant magnetic stirring for four days. After that, 1000 mL of distilled water was mixed into the solution, and then it was vacuum-filtered and dried. The dried materials were put into a beaker of 2 L and then into a microwave at 1000 W for 2–3 min. This method allowed for obtaining expanded graphite, which was then used to synthesize graphene oxide and reduced graphene oxide. The method is well established for the synthesis of expanded graphite [45], and this expanded graphite was utilized instead of graphite powder to achieve rGO.

Similarly to several other authors, we used the well-known Hummers method for the synthesis of GO [46]. All the chemicals were of analytical grade and bought from Sigma Aldrich, and used without the need for any additional purifications. The only difference is that instead of graphitic carbon, we utilized expanded graphite as obtained above from the microwave method. First, we mixed one gram of expanded graphite with 100 mL of H${}_{2}$SO${}_{4}$ under constant magnetic stirring for 15 min. Then, 5 g of KMnO${}_{4}$ was mixed slowly in the beaker, which was kept inside an ice bath container to avoid any explosions due to a sudden rise in temperature due to exothermic reaction. The resultant solution was stirred for 2 h at room temperature.

We performed the synthesis of rGO/Ag nanocomposites as follows. First, 20 mL of GO solution and 80 mL of distilled water were mixed to obtain a 100 mL solution. Separately, 0.01, 0.1, and 1 M aqueous solutions of AgNO${}_{3}$ were prepared by magnetic stirring using 0.16987, 1.6987 and 16.987 g of AgNO${}_{3}$, respectively, and 100 mL of distilled water. Then, GO and each of the AgNO${}_{3}$ solutions were mixed under constant stirring for 1 h. Finally, 5 g of ascorbic acid were mixed slowly and the solution was stirred 1 h to mix the reactants properly and homogeneously. During stirring, 200 mL of water was poured into the mixture and left for one day at room temperature without stirring to complete the reactions. The final product was washed with distilled water and alcohol 2–3 times to remove any by-products or remaining reactants.

### 2.2. Characterization of the Nanomaterials

The structure and microstructures of the synthesized products were confirmed by powder X-ray diffraction (XRD) by an XRD-Shimadzu 6000 (Kyoto, Japan) powder diffractometer (Cu-Ka radiation, 40 kV and 30 mA), a Scanning Electron Microscope (SEM Zeiss, Oberkochen, Germany) at 30 kV, and a Raman spectrometer (Jasco, Tokio, Japan, NRS-4500).

### 2.3. Nanofluid Preparation

The nanofluids were prepared from double-distilled water and rGO/Ag nanocomposites obtained from different molar concentrations of silver (0.01, 0.1, and 1 M). The nanofluid preparation was performed with a two-step ultrasonic method, using a volume of 50 mL of double-distilled water.

After determining the mass of the water volume by using an analytical balance (Boeco, Hamburg, Germany, BAS32), it was poured into a double-jacketed beaker to cool the mixture with a refrigerated circulating bath (JSR, Gongju, South Korea, JSRC-13C) and to maintain the temperature at an approximately constant level, higher than 278.15 K so that no condensate is present on the walls. Equation (1) determines the nanocomposite mass ($m_{np}$), knowing the base fluid mass ($m_{bf}$), and leaving the mass fraction constant (φ) at 0.1 wt.%:

(1) $$m_{np}=\frac{\varphi \cdot m_{bf}}{1-\varphi }$$

The mass of the nanocomposites is determined using an analytical balance (Boeco, BAS32) and then incorporated into the double-jacketed beaker and initially mixed with the aid of a steel spatula. Then, the ultrasonic agitation process is started using an ultrasound probe (Dr. Hielscher GmbH, Teltow, Germany, UP50H). The ultrasonic agitation is performed at a maximum amplitude of 180 µm, frequency of 30 kHz, and power of 460 W/cm${}^{2}$ for 60 min to obtain a homogeneous sample. In stability studies of carbon-based nanofluids, the sonication time was examined, and a time lower than 60 min is required to generate homogeneous and stable samples. Therefore, the prepared nanofluids are considered homogeneous, considering such a sonication time [47,48]. In the end, the nanofluids were prepared using the same rGO/Ag concentration (0.1 wt.%), varying the molar concentration of Ag used to obtain the nanocomposite (0.01, 0.1, and 1 M).

### 2.4. Nanofluid Characterization

The measurement of the nanofluids and the base fluid thermal conductivities required a setup consisting of a double-jacketed vessel, a refrigerated circulation bath, and the thermal properties analyzer (Decagon Device, Pullman, WA, USA, KD2-Pro) with the KS-1 probe. The sample’s thermal conductivity was measured 5 min after ultrasonic agitation to avoid the effect of minimum and maximum local temperatures inside the mixture. Subsequently, the KS-1 probe was inserted entirely and centrally into the sample to reduce the effect of natural convection during the measurement. Then, we waited 2 min before starting the measurements to stabilize the temperature in the probe.

Thermal conductivity measurements were performed for five temperatures, from 283.15 to 303.15 K, with 5 K steps, performing six measurements per temperature level. The average thermal conductivity and standard deviation of water and nanofluids are considered as a representative measure of the property and its associated error, respectively, since the data set is normally distributed with respect to the mean according to the Shapiro-Wilks test at 5% significance level. Finally, the sample was sonicated for 60 min every two measurements to ensure the stability of the nanofluid.

**Figure 1:**
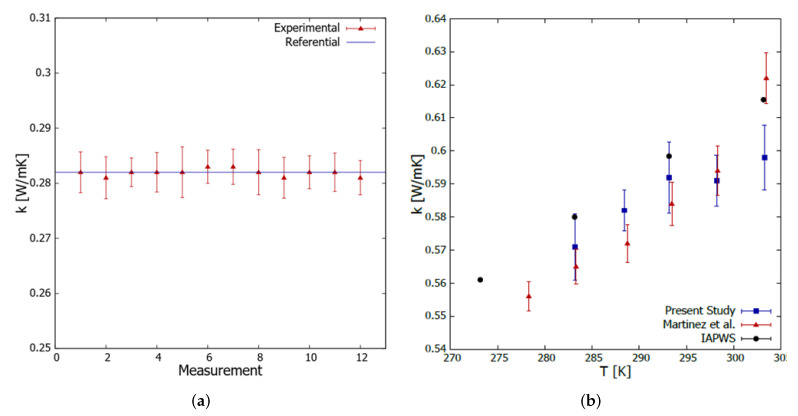
(**a**) Traceability test results of the KS-1 probe with glycerin. The error bars correspond to the KD2-Pro measurement error. (**b**) Assessment of the accuracy of the implemented transient hotwire method. The error bars correspond to the measurements standard deviation.

The traceability process of the thermal properties’ analyzer requires 12 thermal conductivity measurements on glycerin (CAS 56-81-5), whose thermal conductivity is 0.282 W/mK at 293 K. The glycerin is supplied by the equipment manufacturer as standard material for the KS-1 probe. Figure 1a shows the obtained traceability results. A non-parametric Mann-Whitney test was performed to examine whether the experimental results obtained from the thermal properties analyzer differed from the reference value. In this way, it can be assured with 95% confidence that the median of the sample is within the range of 0.281 to 0.283 W/mK, so the median is equal to the reference thermal conductivity of 0.282 W/mK, corroborating the traceability of the equipment.

With respect to the above, the uncertainty in the measurements was calculated at 1%, considering the ratio between the maximum measured thermal conductivity range and the reference thermal conductivity value. The calculated uncertainty agrees with the literature [49]. Moreover, considering water as a reference fluid, the uncertainty value in the entire range is 3.35%, comparing our thermal conductivity measurements with those of IAPWS. However, the error bars in Figure 1a correspond to the KD2-Pro measurement error.

The accuracy test of the implemented transient hotwire method can be observed in Figure 1b, where our water thermal conductivity measurements are compared with those obtained in a recent work [50] and those reported by IAPWS [51]. The transient hotwire method gives consistent results regarding the thermal conductivity of nanofluids [52].

Finally, the thermal conductivity of water and the nanofluids is presented with its respective standard deviation for each temperature level. For the relative thermal conductivity, the error propagation Equation (2) is used to calculate the uncertainty.

(2) $$\frac{\Delta f}{\left|f\right|}=\sqrt{{\left(\frac{\Delta a}{a}\right)}^{2}+{\left(\frac{\Delta b}{b}\right)}^{2}}$$

Here, f=(a+Δa)/(b+Δb), *a*, *b* are arbitrary variables.

The rheological study was conducted using a DV2T-LV Brookfield viscometer with an SC4-18 spindle. The diameter, length, and total length of the cylindrical spindle SC4-18 with a conical tip are 17.48, 31.72, and 35.53 mm, respectively, and the measurements were performed under steady-state conditions. The operating parameters of the viscometer were configured using the “RheocalcT” software. For these measurements, the small sample adapter (7 mL) S4C-13R is used, which provides a double-jacketed vessel connected to a refrigerated circulation bath (Thermoline, Wetherill Park, Australia, BL-20).

The measurements were performed in triplicate for each temperature level in a range from 293.15 to 328.15 K, with steps of 5 K and spindle rotation speeds from 30 to 80 RPM, with steps of 10 RPM. The viscosimeter’s traceability was probed by measuring viscosity of a standard fluid, whose viscosity is 5 mPa s at 298.15 K. The measured values range from 5.15 to 5.19 mPa s, with an average value of 5.17 mPa s, for a confidence level of 95%, where the uncertainty registered was estimated at 3%. The viscosity measurement is reported with the standard deviation registered at each temperature level.

The transient analysis of the thermal conductivity allows for assessing the stability of the nanofluids [53], considering clustering of nanoparticles will have a considerable effect on the thermal conductivity of the nanofluids, which is related to the increase in the average size of the agglomeration, decreasing their magnitude with respect to the time [54]. In this case, 17 thermal conductivity measurements were performed every 2 min, so the duration of each stability study was 94 min. These studies were performed in triplicate at a temperature of 293.15 K.

## 3. Results and Analysis

**Figure 2:**
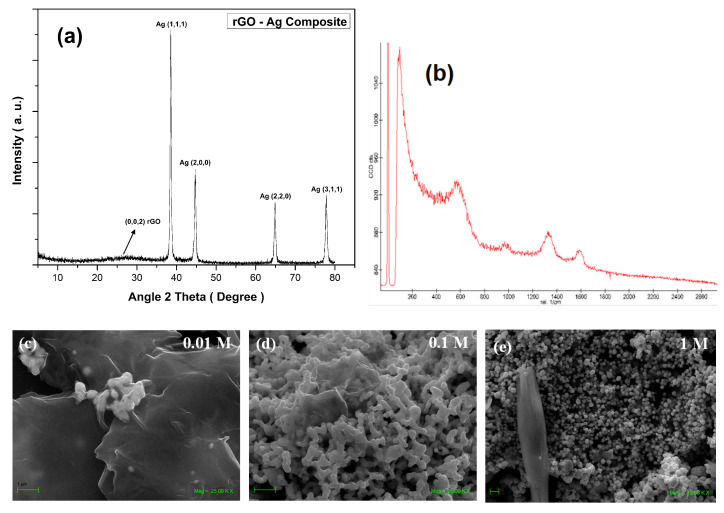
(**a**) XRD peaks of the synthesized rGO/Ag nanocomposites. (**b**) Raman spectra of the synthesized sample. Low and high magnification SEM images of the nanocomposites as-synthesized at (**c**) 0.01 M concentration (**d**) 0.1 M concentration, and (**e**) 1 M concentration of AgNO${}_{3}$.

Figure 2 shows the XRD, Raman spectra, and SEM images of the synthesized rGO/Ag composite. All the XRD peaks as shown in Figure 2a match very well with the silver nanoparticles JCPDS card No 04-0783 [53]. A broad hump between the angles 20 and 35 degrees is the signature of the formation of rGO, but it looks comparatively flat due to the high intensity of the Ag nanoparticles signal. Almost all the different concentrations of silver gave the same spectra, with some intensity variations due to higher concentrations of silver nanoparticles.

Figure 2b corresponds to the Raman spectra of the synthesized sample, where Raman peaks of rGO are visible. Figure 2c–e are magnification SEM images of the rGO/Ag nanocomposites at varied silver nitrate molar concentrations of 0.01, 0.1, and 1 M. Figure 2c is a magnified SEM image of rGO/Ag composite at a molar concentration of 0.01 M, which shows that silver nanoparticles are either in an isolated or cluster form. Figure 2d shows a large number of agglomerated silver nanoparticles and some visible rGO sheets. Higher concentrations resulted in the formation of agglomerated particles rather than isolated particles or clusters.

The highest concentration of silver nitrate, i.e., 1.0 M, resulted in an abundance of larger size silver nanoparticles with large micron-size structures (Figure 2e). rGO/Ag composites at Ag molar concentrations of 0.01, 0.1, and 1 M have particle size ranges between 80 and 150 nm, 100 and 200 nm, and 150 and 500 nm, respectively. These micron-size structures are due to the unreacted salt as a consequence of the insufficient amount of reducing agent for ascorbic acid to react with.

**Figure 3:**
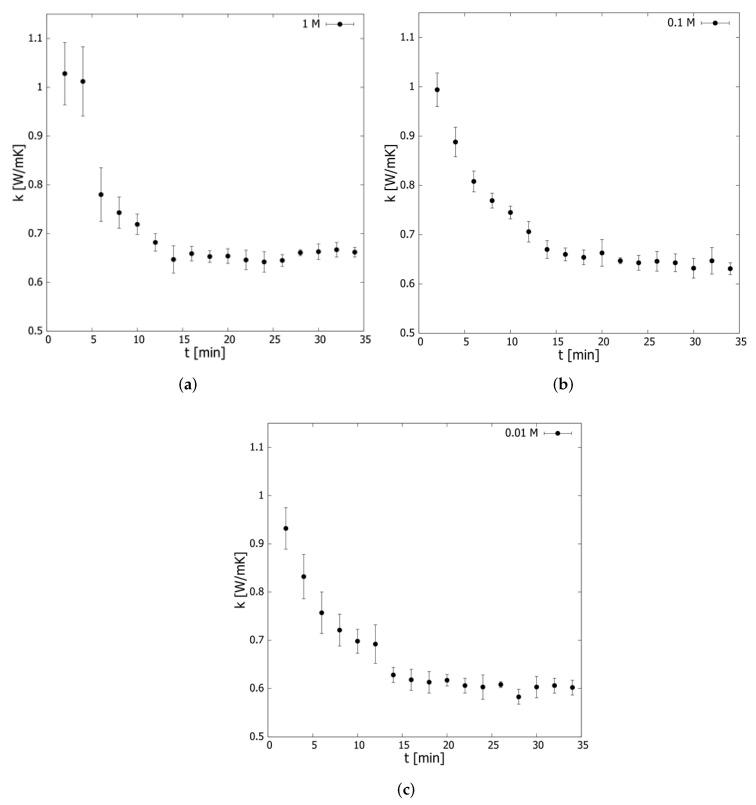
Transient thermal conductivity of nanofluids using rGO/Ag prepared with (**a**) 1.0 M, (**b**) 0.1 M, and (**c**) 0.01 M Ag. The error bars correspond to the measurements’ standard deviation.

The transient behavior of the nanocomposites’ thermal conductivity allows us to evaluate the stability of nanofluids prepared with rGO/Ag (0.1 wt.%) over 34 min at a controlled temperature of 293.15 K (Figure 3), observing an intense decrease in this property between 2 and 6 min of 24.12, 18.71 and 18.78% for the nanofluids prepared with 0.01, 0.1, and 1.0 M of silver, respectively. Two minutes after the sonication process, the nanocomposite particles should have high velocities, leading to an intense convective interaction between the particles and the base fluid (nanoconvection), increasing the thermal conductivity magnitude, as shown in Figure 3.

As time passes, the particles tend to form aggregates, and therefore their velocities decrease due to the viscous effect. The Langevin equation solution predicts that the velocity of the Brownian particles decays exponentially according to a time constant [55]. The set of measurements associated with each nanofluid was fitted to the model proposed by Martinez et al. [56] (Equation (3)), obtaining the magnitude of each model adjustable parameter (Table 1).

(3) $$k\left(t\right)=A\cdot e^{-\frac{t}{B}}+C$$

The results in Table 1 show an inverse relationship between nanofluid stability and Ag concentration. The time constant (B) decreases by 4.24% and 27.96% when the Ag concentration increases from 0.01 to 0.1 M and 1.0 M, respectively, considering a constant concentration of nanocomposite (0.1 wt.%). The model asymptote (C), which corresponds to the stationary thermal conductivity, exhibits a direct relationship with Ag concentration, registering for concentrations of 0.01, 0.1, and 1.0 M of Ag, increases of 0.36, 7.16 and 9.10%, respectively, concerning the base fluid at the same temperature.

**Figure 4:**
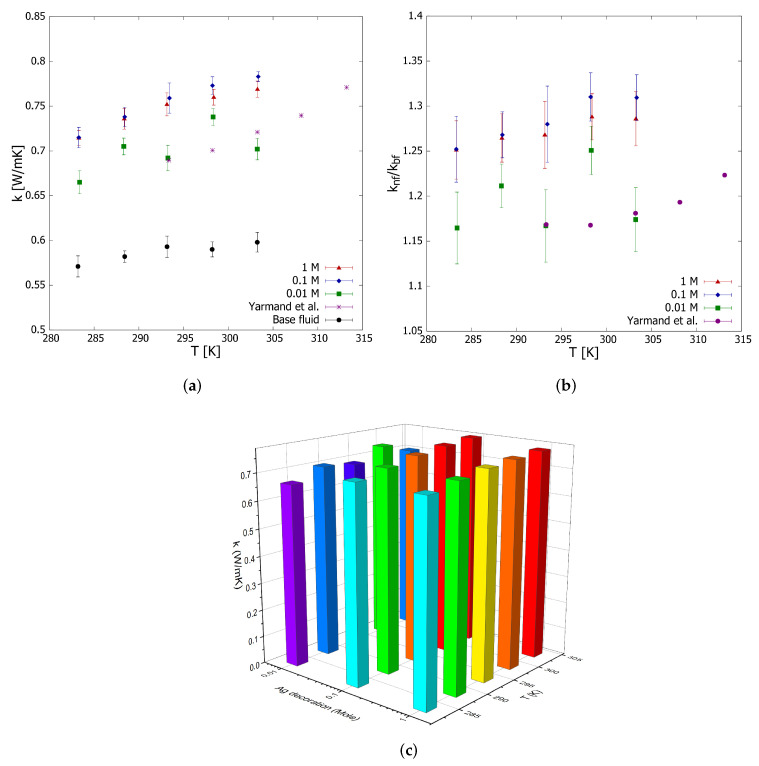
Effective thermal conductivity (**a**), relative thermal conductivity (**b**) and effective thermal conductivity with respect to the Ag decoration and temperature (**c**). The error bars (**a**) correspond to the measurements’ standard deviation and the propagation error (**b**) according to Equation (2).

According to Figure 3, the stationary thermal conductivity values are 14.00, 16.08, and 13.96% lower than those measured at 293 K for concentrations of 0.01, 0.1, and 1.0 M (Figure 4), respectively. These results imply that Ag decoration would improve the thermal conductivity even if the samples exhibit instability.

Similarly, parameter A, which was attributed to the initial improvement obtained from the addition of the nanocomposite, describes an increasing behavior with Ag decoration, increasing by 8.78% (0.1 M) and 43.22% (1.0 M) compared to the rGO/Ag nanofluids with 0.01 M of Ag at a constant concentration of nanocomposite (0.1 wt.%). The transient thermal conductivity measurements were subjected to Shapiro Wilk normality tests with a significance level (5%), revealing that for a confidence level of 95%, the data are generally distributed around the mean. Therefore, a parametric *t*-test is appropriate for evaluating the behavior of the sample.

Following the performed thermal conductivity measurements (Figure 4a), the thermal conductivity is observed to increase on average by 19.22 ± 3.34% compared to the base liquid for rGO/Ag prepared with 0.01 M of Ag and a constant weight fraction of 0.01 wt.%. This improvement amounts to 28.43 ± 2.53% on average, using rGO/Ag with 0.1 M of Ag. However, when using 1 M in the rGO/Ag preparation, when compared to the base fluid, the average increase in thermal conductivity is 26.25 ± 1.53% for both nanofluids at a constant concentration of 0.1 wt.%.

Based on a *t*-test with 95% of confidence, we found that there is no significant difference between the thermal conductivity improvement of both rGO/Ag nanofluids with 0.1 M and 1.0 M. This behavior evidences an asymptotic behavior of the nanofluids’ thermal conductivity regarding the Ag mole fraction used to prepare the rGO/Ag nanocomposite. A 1 M concentration of Ag is relatively large and would even reduce the thermal conductivity of the nanofluid due to the displacement of rGO.

Yarmand et al. [40] prepared aqueous nanofluids of silver-decorated GNP using a 0.05 M AgNO${}_{3}$ concentration. The authors observed a peak in the thermal conductivity enhancement of 22.22% with respect to the pure base fluid over a range of 293–313 K. At a temperature of 303 K, the authors report an improvement of 18.1%. At the same temperature and a lower Ag concentration of 0.01 M, the gain obtained in this work was slightly lower and equal to 17.4 ± 3.02%. Shende and Sundara [57] prepared nanofluids with a nitrogen-doped hybrid structure of rGO and multiwalled carbon nanotubes using DI-water as a based fluid. The authors found a quite similar thermal conductivity augmentation of 17.7% for 0.02 vol.%.

Figure 4b shows the relative thermal conductivity of the analyzed nanofluids concerning temperature, observing in all cases the typical behavior of an increasing trend temperature reported by other authors [9,40], which is explained by the higher kinetic energy of the base fluid molecules and nanomaterial particles. The rGO/Ag nanofluid (0.1 M and 1.0 M Ag) thermal conductivity as a function of the temperature is well described by a linear equation with R${}^{2}=0.967$ [58]:

(4) k=0.30452·T−0.14326

However, at a lower Ag molar concentration, an oscillating behavior in the relative thermal conductivity is observed. Other authors have observed this oscillating behavior as well, studying nanofluids with carbon-based nanocomposites in several mass fractions [59,60].

This behavior can be explained by the intense aleatory motion of the rGO/Ag particles resulting from the nanocomposite–liquid molecule interaction, which is more significant for the samples prepared with a concentration of 0.01 M of Ag decoration due to their comparatively lower mass regarding the 0.1 M, and 1.0 M ($V=\sqrt{(3k_{B}T)⁄m}$). The above is in agreement with the results presented in Figure 3 and Table 1, where the nanofluid prepared with a concentration of 0.01 M displayed higher stability in its thermal conductivity due to its lower deceleration of particle motion.

Although this mechanism enhances the thermal conductivity and stability of the nanofluid, it also increases the convective component of that improvement, which is harmful to the hot-wire technique for thermal conductivity measurements because the method is highly susceptible to convection phenomena in the sample measured. Nevertheless, the validation of this explanation requires more studies to demonstrate the effect of micro-convection on thermal conductivity measurements.

Figure 4c shows a 3D bar graph to better demonstrate the effect of Ag decoration, verifying the asymptotic behavior of thermal conductivity after 0.1 M of Ag and the oscillations mentioned above for the 0.01 M sample.

The rheological study is based on the shear stress with respect to the shear rate (39.6–105.6 s${}^{-1}$). This shear rate range is consistent with our applications of interest, with nanofluids using millichannels as heat sinks with laminar fluids. In this case, the shear rate would give laminar flows in approximately the Reynolds number range of 90 to 250. Moreover, this shear rate range obeys to the fact that at higher shear rates (>100 s${}^{-1}$), rGO/water-based nanofluids show Newtonian behavior [19].

**Figure 5:**
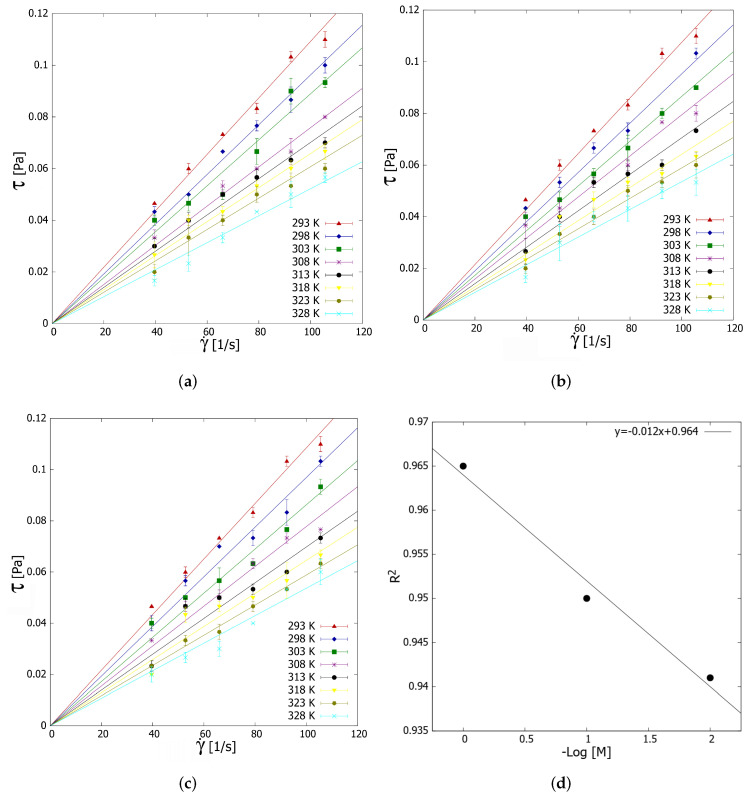
Rheological behavior of graphene nanofluids decorated with (**a**) 1% moles of silver, (**b**) 0.1% moles of Ag, and (**c**) 0.01% moles of silver. (**d**) The correlation coefficient of the Newtonian behavior of the nanofluids with respect to the Ag decoration degree. The error bars correspond to the measurements’ standard deviation.

In general, the results obtained (Figure 5a–c) are described by Newtonian rheological behavior. Thus, the shear stress presents a directly proportional relationship to the shear rate, according to Equation (5):

(5) $$\tau =µ\cdot \dot{\gamma }$$

where τ corresponds to the shear stress, $\dot{\gamma }$ to the shear rate, and µ to the dynamic viscosity. The weight fraction (0.1 wt.%) of the rGO/Ag nanomaterials is not high enough to modify the physical–chemical interaction between water molecules. Several authors have reported the Newtonian behavior of water-based nanofluids prepared with different graphene-based nanomaterials.

Zhang et al. [12] dispersed rGO in DI water and observed a Newtonian behavior at high shear rates. Esfahani et al. [9] showed that GO nanofluids present a Newtonian behavior at higher shear rates (>40 s${}^{-1}$), depending on temperature and concentration. Esfahani and Languri [44] studied water-based GO nanofluids (0.01 and 0.1 wt.%) at 298 and 313 K, reporting a Newtonian behavior for GO concentration of 0.01 wt.% at shear rates higher than 20 s${}^{-1}$. Ranjbarzadeh et al. [61] studied the rheological properties of GO-SiO${}_{2}$ water nanofluids from 293 to 303 K in the range of 0.5–1 vol%, finding that these nanofluids exhibit Newtonian behavior in the shear rates range of 110–245 s${}^{-1}$.

Sadeghinezhad et al. [62] studied rGO-Fe${}_{3}$O${}_{4}$/water nanofluids with a concentration of 0.5 wt.%, which presented a Newtonian behavior above a shear rate of 100 s${}^{-1}$. Using the coefficients of determination (R${}^{2}$), it can be established that Ag decoration strengthens the Newtonian behavior of the prepared nanofluid at a constant weight fraction of nanocomposite (0.1 wt.%). Specifically, R${}^{2}$ presents a linear relationship with the logarithm of the molar concentration of silver (Figure 5d). This behavior suggests that further decoration of rGO with silver leads to a reduction in the shear-dependent viscosity [63], which is explained by the decrease in aggregate formation due to modifying the rGO surface with Ag.

**Figure 6:**
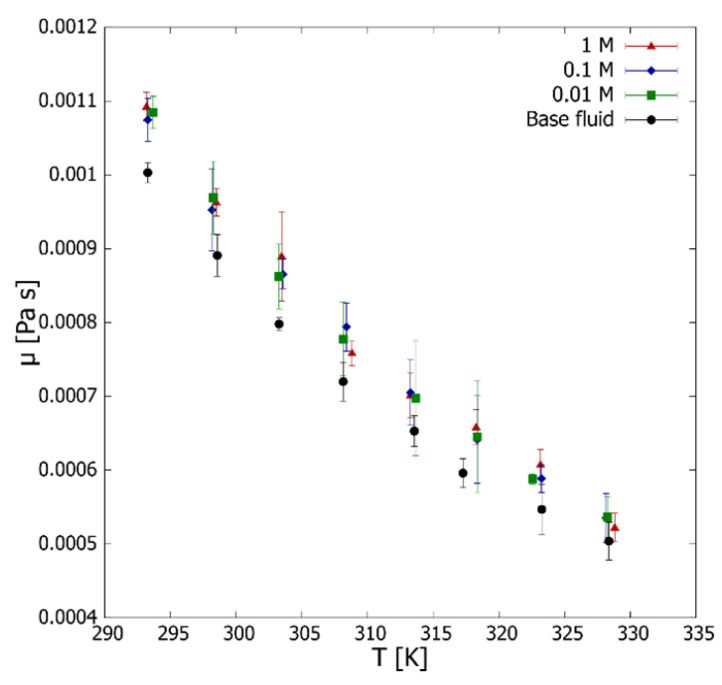
rGO/Ag nanofluid viscosity with respect to the temperature and molar concentration of Ag. The error bars correspond to the measurements’ standard deviation.

Considering water as a reference fluid, we obtained an average uncertainty value of 1.94% for the entire temperature range, considering the ratio between the maximum measured water viscosity range and reference viscosity values [64]. The viscosity behavior of the fluids under temperature was studied utilizing the values obtained in the rheological study (Figure 6). It is possible to observe the decreasing behavior of viscosity concerning temperature, with higher temperatures reducing molecules’ Brownian motion, thermal movement of molecules, and their average speed, which led to a decrease in their intermolecular interactions and their adhesion forces [65].

In general, the addition of 0.1 wt.% rGO/Ag to water does not considerably affect its viscosity. Regarding the rGO/0.01 M Ag nanofluid, an increase in viscosity of 7.76 ± 0.77% is observed concerning the base fluid, which practically did not vary for rGO/0.1 M Ag and rGO/1 M Ag, being 7.78 ± 1.22% and 8.29 ± 2.78%, respectively. Finally, the viscosity of all prepared nanofluids as a function of temperature can be well described (R${}^{2}=0.999$) by a quadratic equation [66]:

(6) $$µ=2.128\cdot 10^{-7}\cdot T^{2}-1.4768\cdot 10^{-4}\cdot T+2.6092\cdot 10^{-2}$$

## 4. Conclusions

We prepared stable rGO/Ag nanocomposite nanofluids with a constant concentration of 0.1 wt.%, using double-distilled water as a based fluid without surfactants. The influence of Ag decoration of rGO was analyzed for three Ag molar fractions (0.01, 0.1, and 1 M). According to the experimental data obtained, the following conclusions are presented:

- The nanofluid thermal conductivity showed increases concerning the base fluid-the most promising being 28.43 and 26.25% for 0.1 and 1 M of Ag, respectively.
- The thermal conductivity presented asymptotic behavior with the molar fraction of Ag. The experimental data showed that, when increasing the molar fraction from 0.1 to 1 M, the thermal conductivity did not improve significantly.
- Based on the time constant of thermal conductivity transient behavior, the increase in the molar fraction of Ag generated instability in the nanofluids studied. The nanofluid prepared from rGO decorated with 0.1 M of Ag corresponded to an optimum, considering the increase of thermal conductivity and its stability.
- The prepared nanofluids presented a moderate increase (<11%) in viscosity compared to the base fluid.
- The nanofluids showed Newtonian behavior at the studied shear rates (40–106 s${}^{-1}$), strengthening such behavior when the molar fraction of Ag was increased.
- Due to their improved thermal conductivity and low viscosity, aqueous nanofluids based on rGO/Ag-decorated nanocomposites are a potential alternative to be used as heat-transfer fluids. Therefore, additional studies should be performed.
- For instance, it is recommended to analyze the effect of the rGO/Ag nanocomposite concentration on the thermal properties and rheological behavior of nanofluids as well as implementations of these nanofluids in heat transfer applications.

## Figures and Tables

**Table 1:** Fitted model parameters for nanofluids prepared with rGO/Ag.

Parameter	0.01 M	0.1 M	1.0 M
A (W/mK)	0.459	0.499	0.657
B (min)	6.208	5.945	4.472
C (W/mK)	0.595	0.635	0.647
S.E. (W/mK)	0.012	0.008	0.032
R${}^{2}$	0.993	0.997	0.968

## Data Availability

Not applicable.
